# Improving equity in respiratory disease outcomes in Africa using data-driven tools: National Institute of Health Care and Research’s (NIHR) EQUI-RESP-AFRICA project

**DOI:** 10.7189/jogh.16.01005

**Published:** 2026-10-23

**Authors:** Igor Rudan, Obianuju O Ozoh, Marie Laurette Agbre-Yace, Godfrey Bigogo, Ziyaad Dangor, Yauba Saidu, Chigozie Uneke, Kadidiatou Raïssa Kourouma, Jibril Mohammed, Michael Budzi, Amour Achy, Yusuf Adelabu, Ariane Adon, Temitope Ayanda, Vicky Baillie, Onyedikachi Chukwu, Amos Enyi, Thecla Ezeonu, Ismail Kalla, Oluchi Kanma-Okafor, Ismaila Karimu, Terry Komo, Winnieclare Muchiti, Nnenna Nweke, Emmanuel Okafor, Mike Osita, Alice Ouma, Shabnam Sheikh, Mbiydzenyuy Ferdinant Sonyuy, Sarah Alexandra van Blydenstein, William Yavo, Harry Campbell, Richard Osei-Yeboah, Davies Adeloye

**Affiliations:** 1 Centre for Global Health, Usher Institute, University of Edinburgh, UK; 2 Nuffield Department of Primary Care Health Sciences, University of Oxford, UK; 3 Green Templeton College, University of Oxford, UK; 4 School of Medicine, University of Lagos, Nigeria; 5 Cellule de Recherche en Santé de la Reproduction de Côte d’Ivoire, Abidjan, Cote d’Ivoire; 6 Centre for Global Health Research, Kenya Medical Research Institute, Kisumu, Kenya; 7 University of Witwatersrand, Johannesburg, South Africa; 8 Clinton Health Access Initiative, Yaoundé, Cameroon; 9 David Umahi Federal University of Health Sciences, Uburu, Ebonyi State, Nigeria.; 10 Institut National de Santé Publique de Côte d’Ivoire, Abidjan, Cote d’Ivoire; 11 Centre Hospitalier Universitaire de Treichville, Abidjan, Cote d’Ivoire; 12 Faculty of Health Sciences, University of Buea, Buea, Cameroon; 13 Reconciliation and Development Association, Bamenda, Cameroon; 14 School of Health and Life Sciences, Centre of Public Health, Teesside University, Middlesborough, UK

**Keywords:** respiratory diseases, Africa, health outcomes, pneumonia, asthma, COPD, pulmonary rehabilitation, CHNRI method, EQUIST tool, PATHS tool

## Abstract

Chronic obstructive pulmonary disease, pneumonia, influenza, asthma, tuberculosis, lung cancer, and COVID-19 are all prevalent in Africa, where they cause a very large burden on the population’s health, but are managed by health systems with a limited capacity for prevention, diagnosis, and treatment. They are also underlaid by substantial inequities in outcomes, with the poorest who have no access to care being affected the most. An interdisciplinary group of academics, policymakers, representatives of non-governmental organisations (NGOs), patients, and civil society are implementing a programme to address key respiratory diseases (RD) in five African countries where morbidity and mortality from RD is very large: Nigeria, Kenya, Cameroon, Cote d’Ivoire, and South Africa. The aim is to build capacity, conduct quality research and an intervention trial, and engage communities and policymakers in order to achieve demonstrable and equitable reductions in morbidity and mortality from RD through evidence-based improvements in prevention, diagnosis, treatment, interventions, and policies. The EQUI-RESP-AFRICA project will map the landscape of respiratory medicine in partner countries through rapid reviews of the available literature and data; engage relevant in-country stakeholders; study patients’ and providers’ preferences and needs; identify research priorities using the Child Health and Nutrition Research Initiative method; and conduct summer schools for capacity building in data science and machine learning/artificial intelligence, in community engagement, and in advocacy for and the use of the Equitable Impact Sensitive Tool (EQUIST) and Pathways to Survival (PATHS) tool. The project will also assess national health system preparedness for emerging infections, identify bottlenecks for early response, assess oxygen capacity and security, study adherence to international guidelines, and conduct qualitative studies to understand barriers to seeking care. It will also explore the feasibility of delivering a pulmonary rehabilitation programme through a randomised controlled trial. Ultimately, the programme will link evidence with policy using the EQUIST and PATHS tools to improve equity in outcomes and identify bottlenecks that lead to morbidity and mortality from respiratory causes. Through high-level meetings, it will prioritise the implementation of equitable interventions that are appropriate to each African context and tailored to patients’ needs. A team of social scientists and NGOs, joined by a team of researchers of diverse backgrounds, will engage communities, stakeholders and policymakers to build capacity through summer schools, and disseminate the results through a dedicated project website, the Journal of Global Health, the Journal of the Pan-African Thoracic Society, collaborating NGOs, and mainstream and social media.

## EQUI-RESP-AFRICA PROGRAMME: BACKGROUND AND RATIONALE

Respiratory diseases (RD), such as chronic obstructive pulmonary disease (COPD), pneumonia, influenza, asthma, lung cancer, tuberculosis, and COVID-19, are highly prevalent in Africa [1–4]. The exposure to risks such as tobacco, outdoor air pollution from industry and traffic [5], indoor smoke from cooking and heating fuels [6], and HIV/AIDS and malnutrition [7] is also high. Despite this large burden, African health systems’ capacity for prevention, diagnosis, and treatment of RD remains very limited [1,8,9]. These systems are diverse, whereby the incorporate both public and private providers, and are regulated to varying degrees, which creates financial barriers, vulnerabilities, and inequity in access to care, leading to diagnoses being often missed and treatments not being based on guidelines [10]. Moreover, RD outcomes are affected by economic status, gender, age, and social exclusion, making them inherently inequitable, with poor and vulnerable being most affected [11]. Lack of standard diagnostics and expert treatment, coupled with weak government support and shortages of funds, equipment and medicines, leads to compromised care for RD [12]. Meanwhile, policymaking and priority setting for RD relies on epidemiological evidence that is scarce or missing.

Gaps in evidence, policies, and care, together with inequitable outcomes, underscore a need for improved response. An interdisciplinary network from the University of Edinburgh (UoE) and five African countries seeks to address those gaps through a complex new programme – EQUI-RESP-AFRICA. We will harness a large body of work conducted by the National Institute for Health and Care Research (NIHR) Global Health Research Unit on Respiratory Health (RESPIRE) at the UoE and in South-East Asian partner countries, and build on the development, validation, implementation, and training for the Equitable Impact Sensitive Tool (EQUIST) and the Child Health and Nutrition Research Initiative (CHNRI) method. We will also pilot and validate the novel Pathways to Survival (PATHS) tool, which is based on decision science that identifies key bottlenecks in intervention delivery.

### Aims and objectives

EQUI-RESP-AFRICA aims to contribute to equitable reductions in morbidity and mortality from major RDs in five African countries through building capacity in epidemiological research and digital health; generating and analysing evidence; conducting interviews, surveys, and trials; engaging communities; involving key stakeholders; linking with policymakers; and improving interventions and policies using evidence-based tools. The objectives of the programme are:

1. mapping the landscape of respiratory medicine in partner countries by gaining a better understanding of the burden of the major RDs, exposure to key risk factors, availability and effectiveness of interventions, and resources available to address RD and equity in RD outcomes;
2. understanding patients and providers’ preferences and needs, as well as barriers to care seeking at all levels of national health systems; and identifying challenges in the diagnosis and management of the major RD and the related adherence to international guidelines;
3. assessing national preparedness for emerging infections, the capacity, accessibility, and safety of oxygen treatments, and bottlenecks to health systems’ emergency response;
4. capacity building in epidemiology research and digital health and AI, in community engagement and stakeholder involvement approaches, and in tools use and advocacy through the summer schools and high-level policy events, with support to PhD and MPH studentships, and with strong community engagement and stakeholder involvement;
5. conducting a multi-country randomised controlled trial (RCT) on pulmonary rehabilitation (PR);
6. implementing the CHNRI, EQUIST, and PATHS tools in defining health research and health intervention priorities, engaging policymakers, and improving policies, leading to more equitable outcomes of major RD.

### Methods

#### Mapping the landscape of respiratory medicine in partner countries and understanding barriers and needs

We conducted a rapid assessment of the data from the five countries’ governments, national and international literature, and public and private facility audits to map the burden of the key RD; assess prevalence and importance of key risk factors; and understand interventions effectiveness, coverage, and costs for the key RDs. We will also conduct qualitative and descriptive research to study barriers to care-seeking in public and private facilities, poor adherence to treatment and lack of follow-up, and limited understanding of patients’ preferences and needs [10], as well as a research priority setting exercise for RDs in Africa using the CHNRI method. Based on the Cochrane guidance for rapid reviews [13], we will review PubMed, African Journals Online, and Google Scholar for evidence on RDs, study exposure to risk factors and characteristics of intervention delivery (*e.g.* cost, effectiveness, and efficiency) by equity strata, and assess the ‘architecture’ of the burden of the key RD by gender, age, and vulnerability of population subgroups. For the interviews studying barriers to healthcare, we will use qualitative content analysis to analyse emerging themes or concepts after describing participants’ characteristics with descriptive statistics. The interviews in local languages will be transcribed using the Jefferson Transcription System, and the deidentified transcripts will be analysed in the Nvivo software. In the cross-sectional observational study, we use descriptive statistics, chi-squared tests, and Student’s *t*-tests to explore differences in adherence across facilities and health providers, as well as construct multivariate models to its determinants [14]. Lastly, we will map medical curriculum and health system components (especially public *vs*. private), identify key actors and stakeholders, and map available government and private resources, documenting their availability and deployment. We will also set research priorities for RD in Africa, and the use of the CHNRI method towards this aim is described in a special section on the tools use (see later) [15].

#### Assessing national preparedness for emerging infections, adherence to international guidelines, and oxygen capacity and safety

Learning from the COVID-19 experience, we will assess the national health systems’ preparedness and response to emerging respiratory infections. To this end, we will review the literature on existing programmes and policies and authoritative media organisations’ reports. After identifying key strengths and gaps, we will design context-based country-wide response strategies to fit the existing national framework, after which we will inform and work with stakeholders: ministries of health, national centres for disease control, immigration and border control officials, health managers, and service providers. Based on our reported challenges with limited diagnostic facilities, expertise and adherence to guidelines for RD [1–3,9], we will conduct a multi-centre, cross-sectional, observational study of public and private health providers using a mixed-method approach, including an adapted Theoretical Domains Framework. We will interview respondents on their understanding, experiences, and challenges in managing major RDs. We specifically address hypoxaemia as a RD management challenge, for which the World Health Organization (WHO) recommends routine measurement and oxygen therapy [16], but in many African countries access to pulse oximetry and oxygen is very low [17]. We will assess the readiness of oxygen delivery systems and oxygen security by documenting the usage, policies, and strategies; assessing designs and gaps; and developing and testing context-specific models for improvement. We will align our approach with other similar initiatives, *e.g.* NIHR RESPIRE Global Health Unit, and ‘Every Breath Counts’ consortium by the Clinton Health Access Initiative and PATH organisations.

We will conduct an independent assessment of national strategies using the WHO’s State Parties Self-Assessment Annual Reporting (SPAR) scores and Johns Hopkins Global Health Security (GHS) index, comparing the two resulting scores with neighbouring countries’ and regional indicators [18]. Learning from the analysis, we will also adapt the WHO/European Observatory resilience framework [18,19]. We will use pre-post intervention design to assess delivery and effectiveness. The selection and sampling of the facilities in which the work will be undertaken will be performed by local researchers based on the understanding of the local context and coordination with local researchers in NIHR RESPIRE Unit, to standardise methodological approaches between South-East Asia and Africa and enable cross-country comparisons and multi-country studies. We will check for adherence to standard guidelines using an adapted Theoretical Domains Framework, designed to assess implementation problems and processes based on medical records and observations in outpatient clinics. We will adapt the WHO/European Observatory resilience framework and relevant contextual domains and analyse collected data from purpose-designed extraction sheets, State Party Self-Assessment Annual Reporting Tool (SPAR) and Global Health Security Index GHS. Structure assessment tools include structured, semi-structured, and e-questionnaires, key informant interviews, observations, and checks in facilities. Descriptive statistics will assess availability, readiness, knowledge, awareness and practice; composite scores will summarise levels of health facility readiness and provider awareness, country-level estimates, and differences in outcomes pre- and post-intervention. For oxygen-related research, we will adapt the Realist Review framework for oxygen assessment [20] to map and test theories and contextual and programmatic factors that can improve the oxygen systems [20,21]. We will conduct interviews with diverse stakeholders to review national oxygen recommendations, and we perform cross-sectional health facility assessment survey to assess oxygen delivery systems and practices (capacity, safety).

#### Capacity building, stakeholder involvement, community engagement, and translation into policy

We conducted a survey of training needs in partner countries and address them using resources available from the NIHR RESPIRE’s platform and the Global Health Respiratory Network. For the first EQUI-RESP-AFRICA school, we focused on training in research methods, statistical analyses, the use of the CHNRI, EQUIST, and PATHS tools, data science and the use of related techniques and approaches (*e.g.* digital epidemiology, big data analytics, machine learning (ML), and artificial intelligence (AI)), and their relevance in global respiratory medicine and public health. The team from the University of Edinburgh consists of a strong group of experts in this field who developed a comprehensive curriculum for knowledge transfer from the UK to five African countries and beyond through collaboration with the International Society of Global Health (ISoGH) and the Pan-African Thoracic Society (PATS) to maximise impact. Two PhD and four MPH studentships are also to be supported within the EQUI-RESP-AFRICA programme.

In the second EQUI-RESP-AFRICA school, experts in community engagement and stakeholder involvement (CSEI) will deliver a comprehensive curriculum which will build capacity in CSEI in order to empower local CSEI initiatives in the five African countries. A high-level meeting with stakeholders and policymakers takes place last to discuss key findings and intervention approaches. It will serve to recommend equitable evidence-based interventions for RD based on the EQUIST and PATHS tools analyses, with support from international organisations – the WHO, the United Nations Children’s Fund (UNICEF), and the World Bank. Through their influence in our partner countries, EQUI-RESP-AFRICA will develop pathways to national ministries of health, promoting equitable interventions for RD. A survey will assess the performance of the summer schools and the high-level meeting. We will trace the impact of the outputs of the meetings through the media, social networks, and the scientific literature, and will study the uptake through targeted interviews. This line of activity creates a cohort of early career researchers and programme experts who act as champions for major RD awareness, prevention, diagnosis, and treatment in their communities and nationally. The CSEI is enhanced through partner non-governmental organisations (NGOs) and new CSEI initiatives arising from this project.

#### RCT on PR

PR is a comprehensive intervention designed for people with respiratory health issues who remain symptomatic or continue to have decreased lung function despite receiving standard treatment [22]. Evidence of improved functional exercise capacity and health-related quality of life as a result of PR exists [22], but less than 3% of RD patients globally have access to PR [23], especially in Africa [24]. The EQUI-RESP-AFRICA programme will explore the feasibility and practicalities of delivering sustainable, context-based PR programmes, seeking to identify the key design considerations for implementation [11]. Home-based and facility-based PR programme is designed and a RCT conducted with a 12-month follow-up to evaluate the effectiveness and completion rates of PR in adults with COPD in the two groups.

The RCT on PR will include adults aged ≥18 years with stable COPD. Participants in both groups will receive the core components of PR, including aerobic exercise training, resistance training, and self-management education [22]. The intervention will involve eight weeks of a home-based PR model, including one home visit and seven once-weekly telephone calls. Participants will receive an educational booklet with illustrated instructions and a diary to keep track of activities performed. The comparator is eight weeks of supervised outpatient facility-based PR model or usual care. The primary outcomes are functional capacity and exercise tolerance, breathlessness, anxiety, or depression. We will assess clinical outcome measures which are reproducible and responsive to change in both groups at baseline, end of rehabilitation, and 12 months later. They are measured by the distance in the Incremental Shuttle Walk Test (ISWT) and Endurance Shuttle Walking Test. In a subsample, we will pilot monitoring through low-cost wearables. We will follow the already established and implemented protocols within the NIHR RESPIRE Unit, to allow comparability and reproducibility between South-East Asian and African countries. Further details on the approach have been described by Habib *et al*. [25] and in the ‘Specific explanations’ section below. All data in a RCT will be analysed by intention-to-treat analysis to assess non-inferiority of home-based *vs.* facility-based models. Differences between groups for change over time will be analysed with linear mixed models, with the relative risk of non-completion determined, and with costing analysis and cost-effectiveness analysis.

### Use of the CHNRI, EQUIST, AND PATHS tools

#### The CHNRI method to set research priorities

The CHNRI method is used to set research priorities in an equitable, democratic, transparent, and replicable way [15]. It employs the principle of crowdsourcing to generate research ideas and score them against a predefined set of criteria, and has been used in >200 published studies led by national governments, funders, and multilateral organisations to set research priorities for different health issues. In the EQUI-RESP-AFRICA, we will collect research ideas from local experts and synthesise a list of questions around research and interventions on respiratory health relevant to the local context. A final list will be scored by local experts – clinicians and researchers – while local stakeholders will weight the criteria. The criteria considered for prioritisation will be typically, answerability, feasibility, timeliness, deliverability, sustainability, potential for burden reduction, and equity. Intermediate scores for each criterion will be computed as the sum of the scores divided by the total number of answers received. The overall priority score, representing scorers’ collective optimism for each idea, will be computed as the weighted mean of the scores for the five criteria (**Figure 1**). We will also calculate agreement statistics between the scorers. Through the implementation of the CHNRI method, researchers, funders, government, and policymakers will easily be able to view the strengths, weaknesses, and relative ranking of each proposed research question.

**Figure 1:**
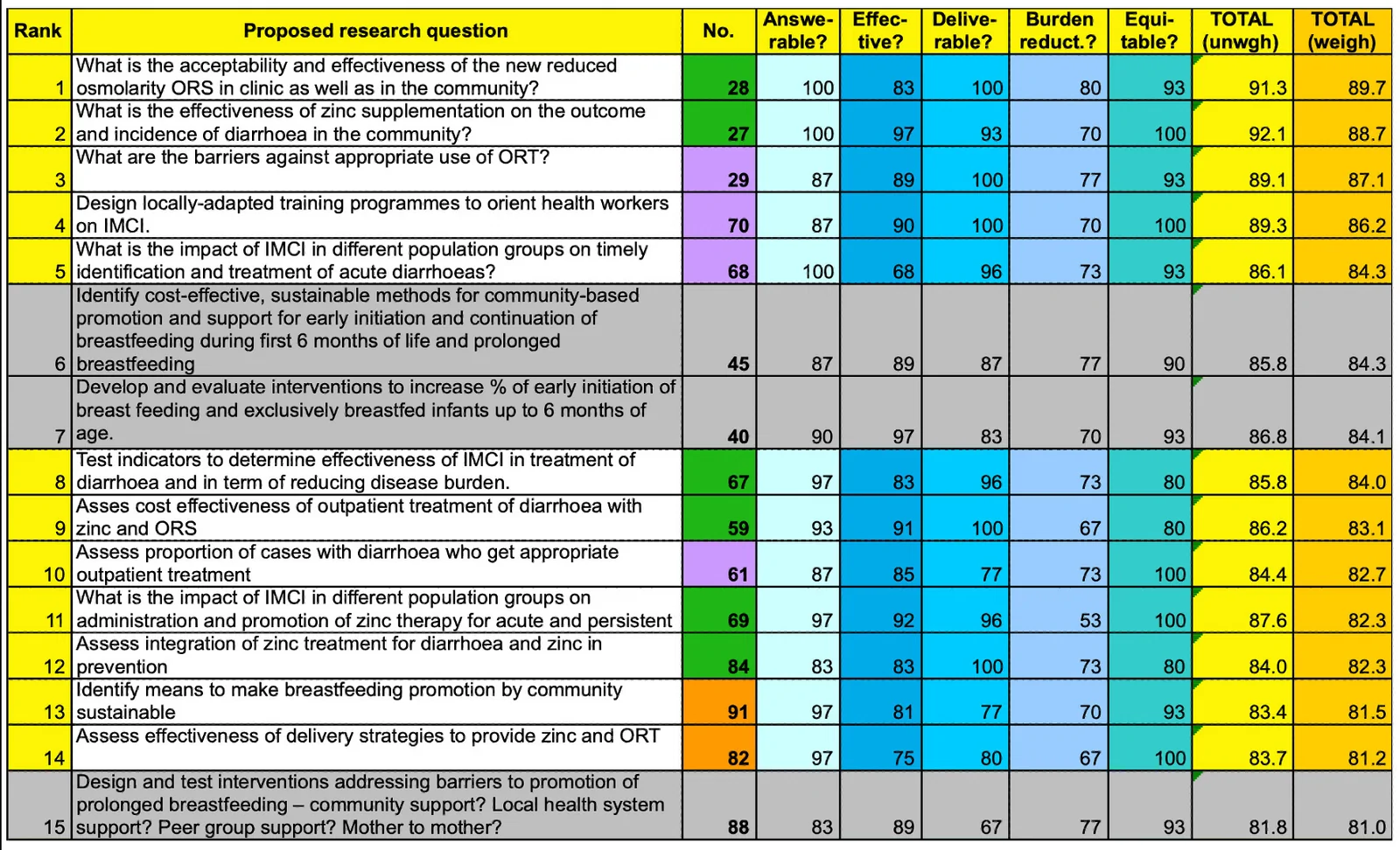
Typical output of the CHNRI tool. The list of proposed research questions usually contains between 100 and 500 questions; only 4 are presented here for practical reasons.

#### The EQUIST tool

The EQUIST tool adds the criterion of the impact on equity to the criterion of cost-effectiveness when prioritising investments in health interventions. This health economics tool and a software/online data platform were developed by UNICEF and the World Bank, and has been deployed to examine the strategies that are both most cost-effective and equitable [26]. It assumes that equity-focused strategies that remove quantifiable bottlenecks can lead to improvements in the coverage of high-impact interventions. The EQUIST has been used to directly influence national health policy and resource allocation in more than 26 African countries, identifying and developing prioritised strategies to address bottlenecks [27]. Presently, there is no agreed-upon approach to prioritising across the spectrum of proven, cost-effective health and development programmes at the national or local level, when immediate needs far exceed the level of funding and resources currently available. Operational teams require a more detailed framework and guidance for systematically comparing and evaluating investment options across the health and development spectrum, while taking specific contextual factors into consideration.

We conduct an EQUIST scenario analysis that helps identify cost-effective and equitable intervention packages for pneumonia, asthma, and COPD, along with the bottlenecks and strategies to address them. We develop a flexible approach that allows country-level teams to derive the optimal mix based on their local information and knowledge. We develop an algorithmic process to assist country teams to objectively select the optimal mixes of interventions for different epidemiological profiles and for the different delivery platforms based on the EQUIST, such as community health workers, outreach, primary healthcare, and so on. The core algorithm of EQUIST (**Figure 2**) takes the decision-making process through the assessments of efficiency of intervention delivery by equity strata, effectiveness, impact, and cost-effectiveness [26,27]. It determines cost-effectiveness and the impact on equity of competing interventions, and identifies and promotes interventions that can equitably reach vulnerable populations within available resources.

**Figure 2:**
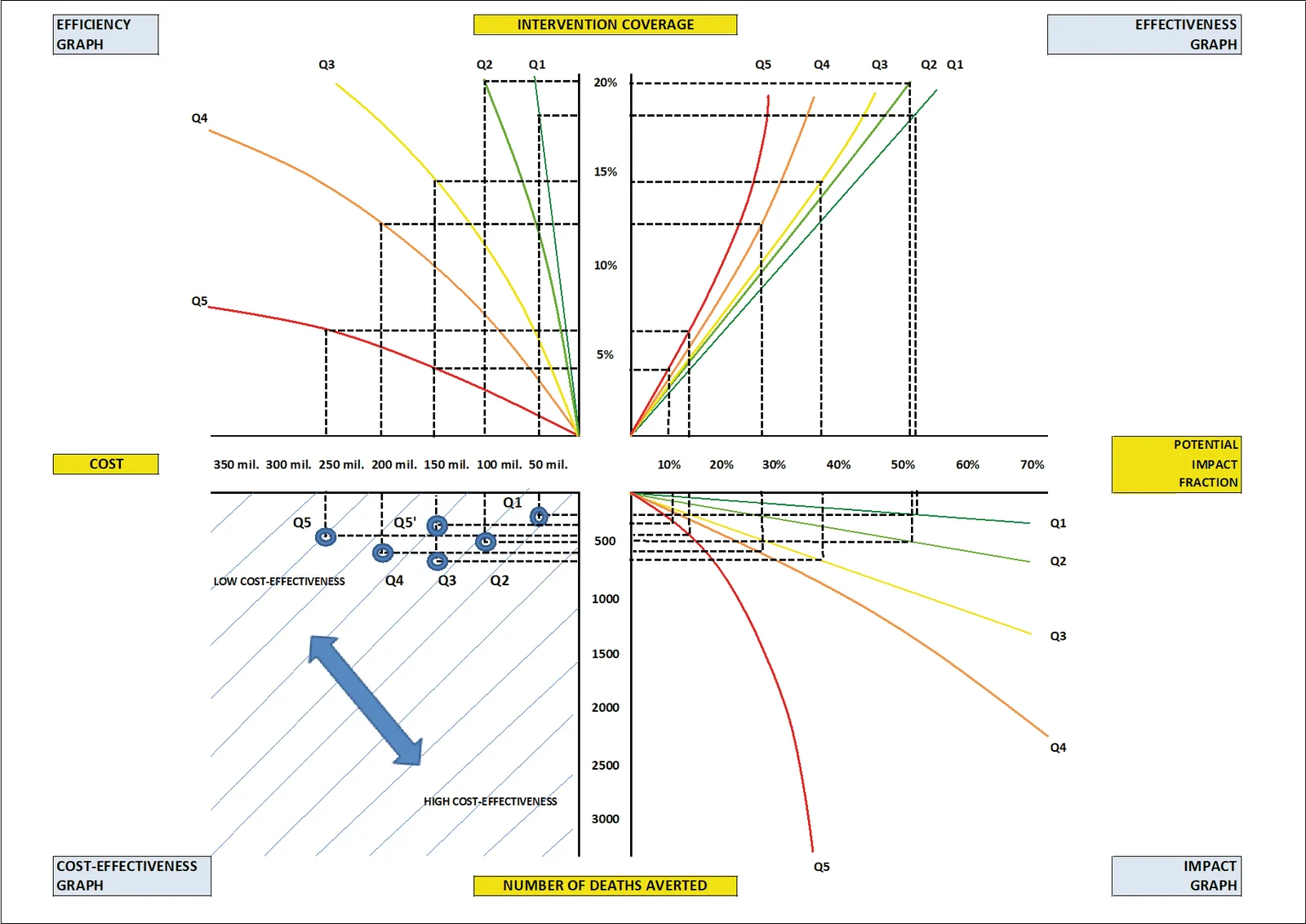
The EQUIST approach to prioritising the effectiveness of interventions. Source: Chopra *et al*. [28], published under CC BY 4.0 license.

#### The PATHS tool

The PATHS tool identifies key bottlenecks in health systems’ care provision that are responsible for most of the severe morbidity and mortality burden. It employs an epidemiological model using decision trees, available evidence and expert opinion [29], thus visualising the ‘architecture’ of mortality in the population by following the entire population cohort over a certain period of time. It explains how initially healthy persons progress through health systems to lethal outcomes at the end of the specified time period. The PATHS has the capacity to expose key bottlenecks in health systems that are the root cause to the severe outcomes and/or deaths, and the removal of which would have the largest potential to improve those outcomes. It especially brings to attention the causes that are associated with poverty and underprivileged groups. For the PATHS tool, we develop illustrative models and pilot them based on the estimated number of cases and deaths from pneumonia, asthma, and COPD (**Figure 3**).

**Figure 3:**
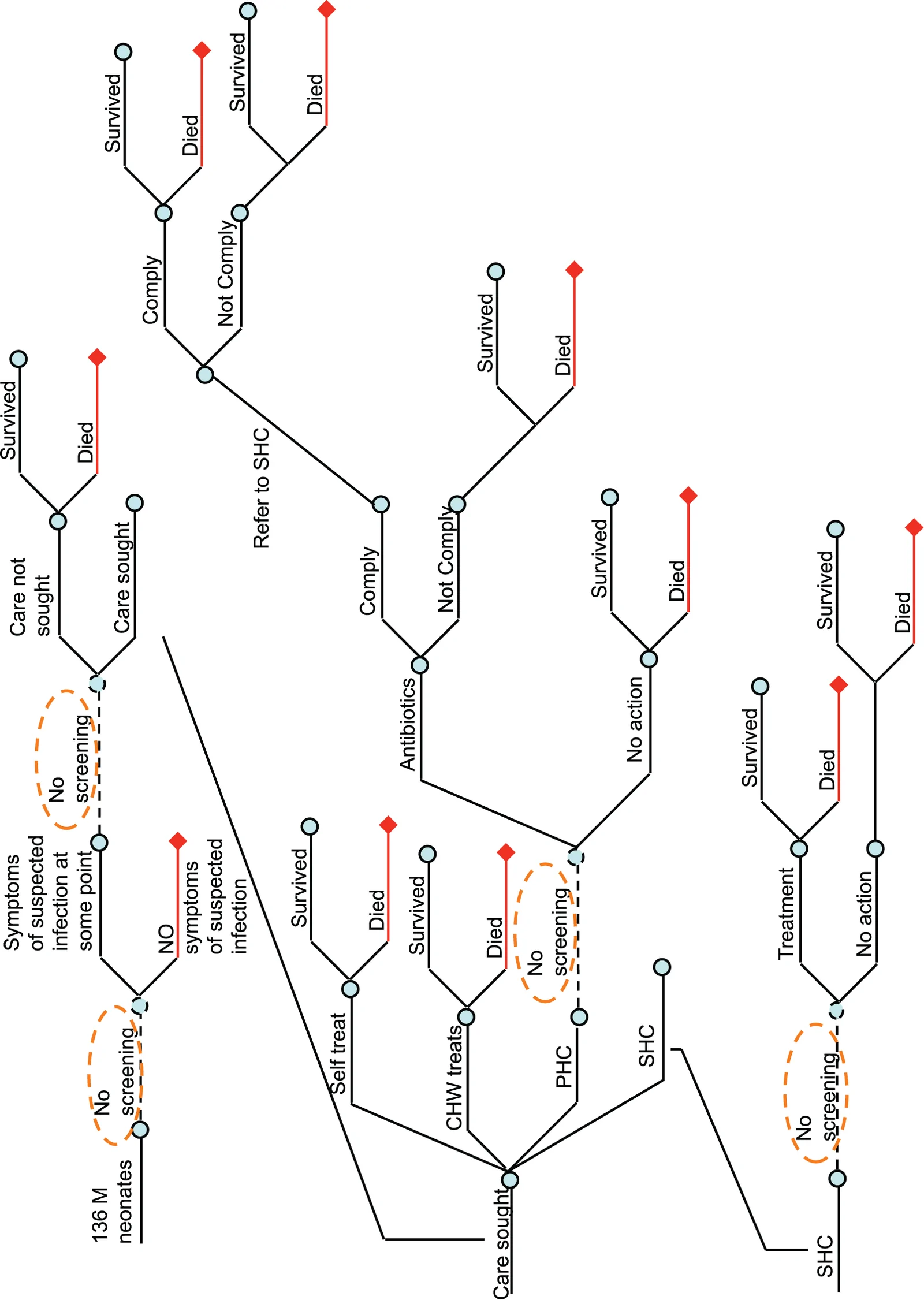
Basic epidemiological model showing possible pathways to deaths from neonatal infections globally and four points of possible interventions with diagnostic tests. Source: Rudan *et al*. [29], published under CC BY 4.0 license. CHW – community health worker, PHC – primary healthcare physician, SHC – secondary healthcare.

The PATHS tool also determines bottlenecks to implementing interventions and providing care and exposes their negative effects. In doing so, it identifies interventions that can equitably reach vulnerable populations within available resources and directly translates and communicates these results to key stakeholders and policymakers, together with the recommendations based on the CHNRI method and the EQUIST tool. This provides data-driven, replicable, and comprehensive policy advice to the key stakeholders in partner countries, in which they can take part and ownership themselves and engage local communities.

### Structure of the EQUI-RESP-AFRICA Collaboration

The Centre for Global Health (CGH) at the University of Edinburgh, co-led by the co-principal investigator of this programme, Professor Igor Rudan, is a WHO Collaborating Centre for Population Health Research and Training. It is part of the Usher Institute, which excels in data science and digital health in medicine and has a strong international reputation in respiratory health research. CGH produced hundreds of impactful publications and several WHO reports and guidelines and has multiple ongoing programmes – *e.g.* NIHR RESPIRE, AUKCAR, RESVINET, PROMISE. The core team in Edinburgh is supported by the two key administrators, Ms Stephanie Scott and Ms Natalia Meyer-Gomez, and by a postdoctoral researcher, Dr Richard Osei-Yeboah, with a number of other active Edinburgh-based researchers also being part of the larger team. The key partner in Africa is the Lagos University Teaching Hospital and PATS Methods in Clinical, Epidemiological and Operations Research (MECOR) initiative, which trained >300 students from 22 African countries over 15 years, many of whom have become regional research leaders and policymakers, and which collaborates with the future Respiratory Disease Observatory for Africa and the Journal of the PATS. At the University of Lagos, the team leader is Professor Obianuju O Ozoh, who is also a co-principal investigator of the EQUI-RESP-AFRICA programme. Other partners in Africa include David Umahi Federal University of Health Sciences in Nigeria, with Professor Chigozie Uneke a local leader; then, Kenyan Medical Research Institute in Kenya, with the national representative Dr Godfrey Bigogo; *Cellule de Recherche en Santé de la Reproduction en Côte d'Ivoire* in Cote d’Ivoire, led by Dr Marie Laurette Agbre-Yace, and supported by Dr Raissa Kourouma, who coordinates the CESI activities on EQUI-RESP-AFRICA programme. The country representative in Cameroon is Dr Yauba Saidu, of Clinton Health Access Initiative, who coordinates EQUI-RESP-AFRICA activities on oxygen research. The national lead in South Africa is Professor Ziyaad Dangor, who also leads laboratory work. The EQUI-RESP-AFRICA also has an international steering committee, which serves as an external advisory group to advise and assist the programme. It consists of the leading global health authorities on respiratory health: Professor Heather Zar (chair), Professor Olufunke Adeyeye, Professor Sally Singh, Dr Shamim Qazi, Dr Jamie Rylance, and Dr Bruce Kirenga.

### Specific considerations

**Table 1** shows more specifically which diseases are addressed by which partner, through which proposed research, and at what level of health system. They are grouped into two clusters: paediatric (childhood pneumonia, influenza, and asthma) and adult (COPD, asthma, pneumonia and long COVID). Paediatric diseases are studied at all six sites, while adult diseases are studied at four sites. Then, barriers and bottlenecks, as well as management challenges, are studied at both the community and facility level at all six sites. Safety and availability of oxygen treatment are studied at four sites, while the RCT on pulmonary rehabilitation are conducted at three sites.

**Table 1:** Present capacity of the six partner institutions in five partner countries to conduct research on the major respiratory diseases at the community and facility level in the four areas of study: barriers and bottlenecks, management challenges, oxygen safety and availability, and RCT on PR

Partner institution		Nigeria (Lagos)	Nigeria (DUFUHS)	Cote d’Ivoire (*CRESAR-CI*)	Cameroon (CHAI)	Kenya (KEMRI)	South Africa (WITS)
Respiratory diseases addressed*		**Paediatric and adult**	**Paediatric and adult**	**Paediatric**	**Paediatric**	**Paediatric and adult**	**Paediatric and adult**
Research on	Level	Capacity	Capacity	Capacity	Capacity	Capacity	Capacity
Barriers and bottlenecks	Community	Yes	Yes	Yes	Yes	Yes	Yes
	Facility	Yes	Yes	Yes	Yes	Yes	Yes
Management challenges	Comm	Yes	Yes	Yes	Yes	Yes	Yes
	Facility	Yes	Yes	Yes	Yes	Yes	Yes
Oxygen safety, availability	NA						
	Facility	No	Yes	Yes	Yes	Yes	No
RCT on PR	Community	Yes	No	No	Yes	No	Yes
	NA						

CHAI –, *CRESAR-CI* – *Cellule de Recherche en Santé de la Reproduction de Côte d’Ivoire*, DUFUHS – David Umahi Federal University of Health Sciences, KEMRI – Kenya Medical Research Institute, NA – not applicable, PR – pulmonary rehabilitation, RCT – randomised controlled trial, WITS – Wits Health Consortium.

*Paediatric diseases include childhood pneumonia, influenza, and asthma; adult diseases encompass chronic obstructive pulmonary disease, asthma, pneumonia, and long COVID.

In conducting these lines of research, we will follow protocols and approaches that have already been developed by the NIHR RESPIRE Global Health Unit for Bangladesh, India, Pakistan, and Malaysia. In this way, we will expand the knowledge base on these issues to two world regions where the needs are greatest and ensure comparability of the findings across several countries. The longer-term aim is to continue to expand the number of low- and middle-income countries (LMICs) in which this research has been conducted using comparable set of methods and approaches, thus maximising the value for investments in this research and the impact of the results. The RESPIRE-2 Global Health Unit has already expanded to three further South-Asian countries (Sri Lanka, Indonesia and Bhutan). With five further countries in Africa adopting the same methodological approaches within EQUI-RESP-AFRICA, the two NIHR-funded programmes now include a total of 12 countries with intense multi-dimensional research, capacity building and community engagement programmes based on comparable set of methods and approaches that are increasingly refined. Below we provide further details on the methods that will be used to address key research aims, based on the experiences from the NIHR RESPIRE Unit’s programme.

Methods to identify patient and provider needs have been developed and reported by the NIHR RESPIRE Units in India [30] and Malaysia [31]. Barriers are identified through a systematic review of interventions that address limited health literacy, conducted by the NIHR RESPIRE Unit [32], while stakeholders’ perspectives on barriers and challenges in Malaysia are also methodologically addressed [33]. Methods to study challenges in diagnosis and management of major RDs within the NIHR RESPIRE Unit were reported in a systematic scoping review of methodological approaches and outcomes for surveys [34]. Then, NIHR RESPIRE also provides a systematic review protocol of methodologies of surveys [35] and methods for studying the implementation of international management guidelines [36]. Methods to study oxygen accessibility and safety were also developed by the NIHR RESPIRE Unit to review national oxygen recommendations and conduct formal consultations with experts to finalise survey instruments, and there is already a well-developed case approach in Cameroon, within the EQUI-RESP-AFRICA programme, which will serve as the basis for research in other partner countries in Africa [37,38]. The NIHR RESPIRE Unit also developed methods to plan a RCT of PR based on a systematic review on the components, delivery, and effectiveness of PR [25]. It also offers methods to engage communities and stakeholders, both in India [39] and in four countries simultaneously (Bangladesh, Pakistan, India, and Malaysia) [40].

### Approach to creating equitable and sustainable partnership

The EQUI-RESP-AFRICA programme promotes values based on respect, reciprocity, and sustainability. It builds on the excellent working relationships that we have already established between partners working on the Summer School for Global Health Economics and Capacity Building in the EQUIST tool in Edinburgh in 2019. The management structure is based on co-leadership within all the key roles by both LMIC and UoE staff, alongside with an expert from an international organisation. The aim is to gradually shift the balance of responsibility and power to partners from LMICs over the course of the project. All major decisions on the EQUI-RESP-AFRICA are made by the group management committee attended by all staff in key roles monthly. Commitment to gender equity is apparent in all EQUI-RESP-AFRICA roles, with female members being equally represented in the management roles.

EQUI-RESP-AFRICA invests in enabling and promoting Patient and Public Involvement (PPI) and community engagement and involvement, as well as working with ministries and other policymakers in partner countries. Workshops will be held in countries to introduce and support national health policy and localised district level programmes to develop investment cases involving communities, policy makers, patients, and other stakeholders. Our team of social scientists and NGOs champion engagement activities, building on the examples of the PATS MECOR initiative, a network of community health NGOs, including FENOSCI (in Cote d’Ivoire), C21 (in Nigeria), CACSNA (in Cameroon), and AAOKENYA (in Kenya), as well as community advisory boards in South Africa. We will survey training needs in partner countries and respond using resources already available from the NIHR RESPIRE Unit’s platform and transferring South Asian experiences to Africa.

The CHNRI method-based research priority setting exercise ensures that specific research projects in EQUI-RESP-AFRICA are based on topics proposed and prioritised by African partners: academics, policymakers, NGO members, communities, service providers, and patient groups. We aim to support them all in identifying key needs, bottlenecks, and gaps in knowledge and expertise using the CHNRI method. We will provide support to PhD and MPH students from Africa to address some of the identified research priorities in their work. We will also hold regular workshops on local needs and priorities to provide a platform to national champions of stakeholder engagement, and will ensure that their views inform research priorities in EQUI-RESP-AFRICA. Specifically, for the implementation of the EQUIST tool, ministries will be supported by the local World Bank, UNICEF, and WHO offices, academic institutions, building their capacity in both systematic reviews and EQUIST-based analyses. Local core groups comprising of senior health policy strategists, senior data specialists, and community and patient representatives will take ownership of data entry, including local indicators, parameters, and bottlenecks that exist within their context. This further allows them to initiate their own analyses and to generate and upload new datasets.

### Training and capacity strengthening in LMICs

One of the overarching goals of EQUI-RESP-AFRICA is to enable knowledge translation between the UoE-based researchers on one side, who are internationally leading in epidemiology, statistical methods, tools development and application, cost-effectiveness and equity analysis, data science, big data analytics, ML and AI, qualitative research methods, health policy and systems research, CSEI methods and clinical skills, and the early-to-mid-career researchers in African partner countries, who are expected to champion the implementation of this knowledge and skills in African respiratory medicine. Edinburgh-based experts will work alongside their African counterparts to generate knowledge and policy advice. The EQUI-RESP-AFRICA particularly promotes South-to-South partnerships and peer support in training and secondments for skills development.

Through its annual schools, EQUI-RESP-AFRICA will build expertise in all aspects of data science and digital health, research methods for knowledge synthesis, surveys and RCTs, for the use of priority setting tools, health systems and policy research, and in designing and conducting small formative studies. Then, it will train African researchers in cost-effectiveness and equity analysis through the use of the EQUIST tool, bottleneck analyses through the use of the PATHS tool, big data analytics, and ML and AI-based methods. Wherever possible, EQUI-RESP-AFRICA promotes context-relevant methodologies by engaging local methods expertise. Building on NIHR RESPIRE Unit’s work, EQUI-RESP-AFRICA will promote the use of approved and/or novel methods through collaboration with methodology experts, including through networking with The Global Health Respiratory Network and the International Society of Global Health. These activities are expected to result in improved data management, documentation, and quality assessment that is scalable to other African and LMICs.

EQUI-RESP-AFRICA will also promote improved data management strategies, policies, operating procedures, training, and assistance through specific guides. It will advance open data sharing policies and foster end use of data, establishing services and systems for safe, secure data storage, and wider data discoverability. EQUI-RESP-AFRICA data champions will work to ensure compliance with General Data Protection Regulation/Data Processing Agreement and local data laws and policies. Data sharing knowledge exchange builds on the NIHR RESPIRE Global Health Unit to ensure legacy in developing expertise and capacity in global South. These activities are expected to lead to the development of a wide range of research skills, such as grant writing, integration of PPI/CESI, data skills, qualitative methods, evidence synthesis, priority-setting tools use, digital health and AI, but also expertise in governance and financial management.

Patient advocacy and empowerment through working with community leaders will also be strongly supported. We will develop patient advocacy training and engage with a wide range of stakeholders with varying educational/training needs, as well as consult African partnering NGOs to develop and rank a list of possible learning objectives. We will build patient’s and carer’s confidence and skills in RD (self-)management, enhance the media’s, patients’ and carers’ lay knowledge and understanding of RDs, improve healthcare professionals’ technical knowledge and understanding of RDs and clinical skills, and build their confidence in implementation and knowledge translation. We will also enhance policymakers’ and community leaders’ lay knowledge and understanding of RDs and the use of data-driven tools in policy formulation.

EQUI-RESP-AFRICA also builds on the array of existing educational resources developed by RESPIRE Unit’s partners in South-East Asia and tailors them to the African context and to specific groups of African stakeholders. Building on UoE’s track record in delivery of online learning, we will create online, open access e-learning platforms, bringing together learning and teaching materials addressing our priority learning objectives. This will serve both as a learning hub and as a resource for educators and a repository for educational materials that can be adapted to different needs. Our vision is to create a platform that can be accessed by clinical educators in LMICs tasked with designing workshops on, for example, childhood asthma for community health workers, or similar needs. We will work to identify EQUI-RESP-AFRICA partner institutions that can deliver certificated, degree-level training targeting our learning objectives and ensure EQUI-RESP delivers formal qualifications.

### Management and governance

The management model comprises a group management committee representing each partner and advised by an international steering committee (**Figure 4**). The first directs, monitors, and reports progress towards achievement of EQUI-RESP-AFRICA’s aims and commitments, ensuring that all decisions are reached jointly, governance procedures are followed, ethical and sponsorship approvals are obtained, best practice is followed in research conduct, and safeguarding procedures are in place. Managerial procedures established in EQUI-RESP-AFRICA include clear demarcations of responsibilities and reporting and promote equal and respectful relationships.

**Figure 4:**
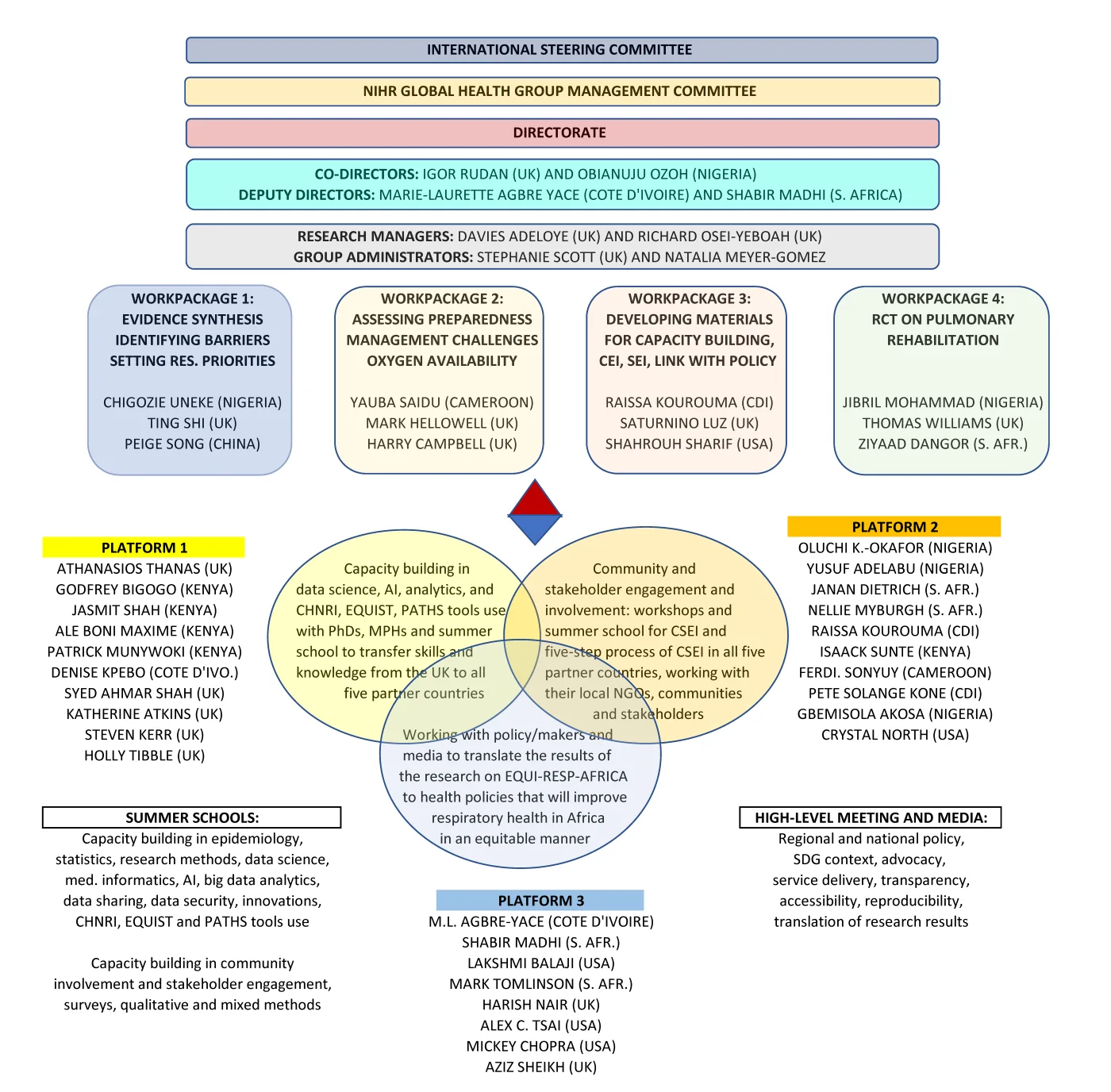
Management and governance structure of the EQUI-RESP-AFRICA programme.

The four work packages and three platforms of EQUI-RESP-AFRICA (**Figure 4**) are jointly led by one LMIC and one UoE-based topic expert, and one external topic expert who is affiliated to an external international organisation active in global health, such as the WHO, UNICEF, World Bank, or universities such as Harvard, Zhejiang, or Stellenbosch. The teams working on the four work packages and three cross-cutting platforms meet regularly to exchange information, build capacity, monitor progress and manage risks, and to facilitate dissemination and translation to policy. The lessons from the NIHR RESPIRE Global Health Unit were that, although the cross-cutting platforms were effective on their own, there was suboptimal collaboration between them, and translation of findings of the programme into policy and practice was linked to individual, specific subprojects. To improve the synergy of platforms and maximise their impact, EQUI-RESP-AFRICA will establish a cross-platforms coordination, comprising of platform leads who meet regularly to enhance EQUI-RESP-AFRICA’s translational work across platforms, promote synergies, and improve links of all platforms with all work packages. We aim to identify every opportunity to enable the new knowledge to have impact on the community and engage all stakeholders and policymakers, and that the realised impact is both equitable and sustained. This coordination will also plan how to transfer contributions on respiratory health towards achieving national SDG goals. Our CESI group builds a network of national community engagement and stakeholder involvement champions, who engage with local communities, provide feedback and communicate their needs to the group management committee.

The annual scientific meeting of the EQUI-RESP-AFRICA programme is the most important regular checkpoint that brings together all the investigators and support staff working across EQUI-RESP-AFRICA, and the international steering committee. It will include a combination of plenary sessions, work package sessions, and platform specific sessions, through which it will showcase activities, share knowledge, plan joint initiatives in respiratory health, and obtain feedback from the international steering committee. We will also provide opportunities for early career researchers to present their findings, paying particular attention to gender equity.

### Dissemination

Our dissemination strategy communicates project results among different stakeholders to promote community awareness and engagement and impact on policy and practice. This is achieved through multiple coordinated approaches:

- development of EQUI-RESP-AFRICA’s website to disseminate findings and raise awareness of respiratory health among the scientific community, policymakers, health professionals, and the public;
- strong support and promotion of the project’s website through webpages and groups on social networks: Facebook, Twitter, and LinkedIn (with further support from the ISoGH and PATS);
- creating a network of links between the EQUI-RESP-AFRICA website and social network sites and other websites and social network sites focused on respiratory health and/or African health systems and challenges;
- publication in open-access scientific journals and sharing the publications timely on the website and social media;
- presentations at national and international meetings with livestreaming on social media and the website;
- organisation of dedicated EQUI-RESP-AFRICA meetings to share best practices, and their filming and sharing on YouTube;
- development of project posters, slideshows, videos, and newsletters and their sharing through social networks;
- preparation of press releases and engagement with media, especially mainstream and social media in Africa;
- strong synergies with the existing consortia and initiatives in Respiratory Health (*e.g.* Global Health Respiratory Network, PATS, ISoGH) and national and relevant regional professional associations;
- strong engagement with communities and patients to ensure sustainability;
- strong linking with ISoGH and its dissemination channels, as well as the Journal of Global Health’s group of journals.

### Impact

EQUI-RESP-AFRICA outputs will achieve impact through the following routes (**Figure 5**):

**Figure 5:**
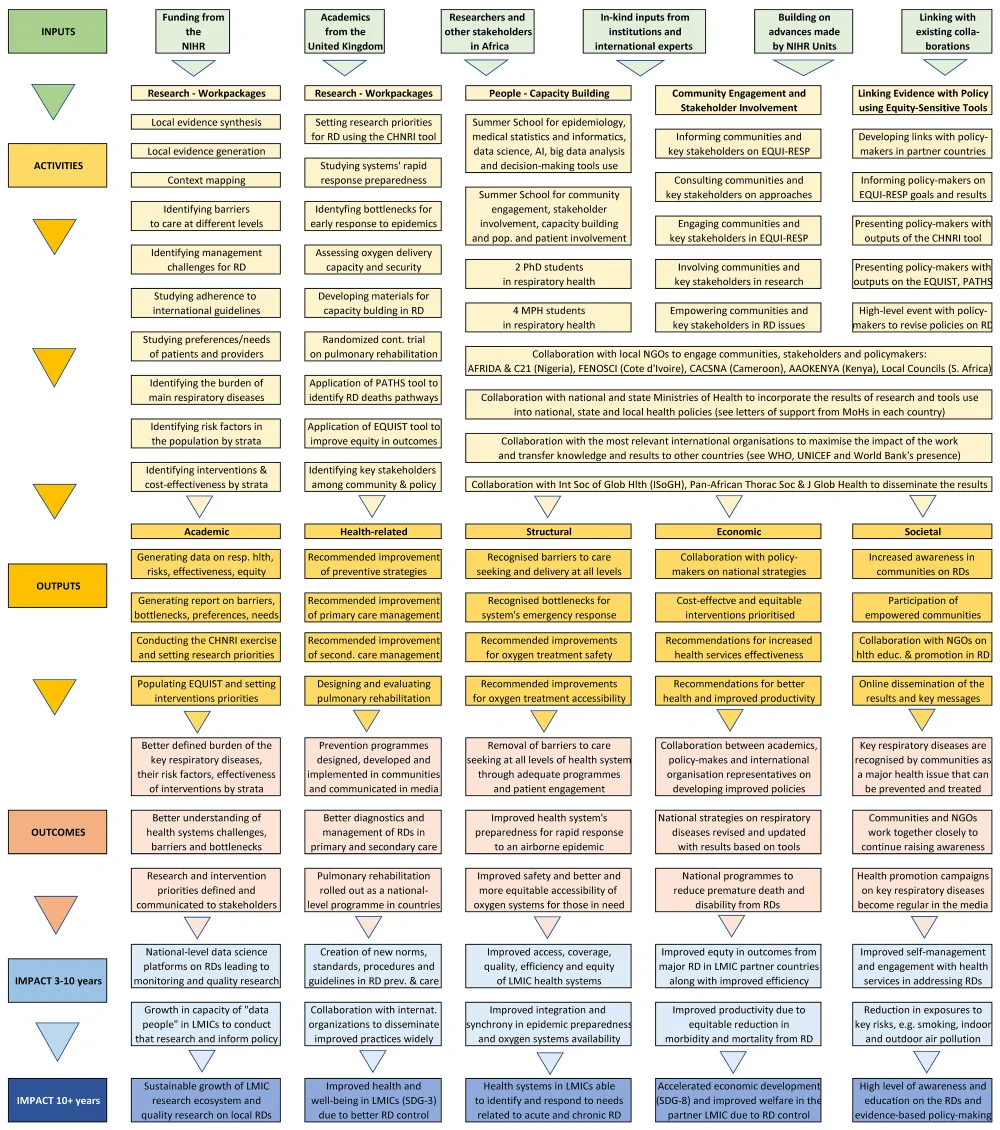
Flow chart of the EQUI-RESP-AFRICA programme. External influences are expected to affect the flow, including changes in the social, political, economic, environmental, technological, legal, demographic, cultural, and other contexts. Multiple interactions between different streams are expected to enhance the resulting impact, such as community engagement and stakeholder involvement, coordination between partners, South-South collaborations, and engagement with other funders and existing and emerging programmes.

- knowledge exchange on epidemiology, data science, digital health, and AI;
- development of effective new or improved interventions, products and approaches, and implementation processes;
- identifying and testing new ways to improve the effectiveness of health services and health policy through implementation of new knowledge from EQUI-RESP-AFRICA work packages and platforms;
- promoting CESI as an integral part of health research and building capacity in communities – *e.g.* identifying and empowering patient advocates who can influence policy and practice;
- academic impact through open-access publications and presentations at (inter)national meetings;
- education and capacity building of a diverse range of stakeholders, including local communities;
- promotion of open data science principles to share new knowledge and underpinning data and promote its reuse;
- health-related impact through creation of new norms, standards, procedures and guidelines;
- preparation of materials based on tools use (CHNRI, EQUIST, PATHS) which will improve local, national and international health policies and make them more equitable;
- economic impact through increased health services effectiveness, improved health, quality of life, and productivity;
- societal impact on local communities to raise their awareness, as well as to improve self-management and engagement with health services.

Given the very large and increasing RD burden, we urgently need population and individual-level interventions which will be effective, feasible, and equitable in African settings if Sustainable Development Goals and universal healthcare targets are to be met locally [41–43]. In addition, respiratory health problems due to tobacco smoking and exposure to indoor and outdoor air pollution, and preparedness for emerging respiratory infection threats, are the leading international health priorities [44]. The EQUI-RESP-AFRICA team have an established track record of their research having real world impact, as reflected in their impact case studies on both respiratory infections and EQUIST tool implementation being included in both the UoE Research Assessment Exercise REF2014 and REF2021 submissions, as well as the University of Edinburgh Principal’s Medal Award for Exceptional Service to Professors Igor Rudan, Harry Campbell, and Harish Nair in 2019 for their global work on pneumonia and EQUIST implementation. This work has led to changes in international policy and investment and has contributed to reductions in global child mortality, particularly from pneumonia. We expect that implementing the flowchart in **Figure 5** consistently over at least a decade would lead to further reductions in morbidity and mortality from RD in the five African partner countries.

## CONCLUSIONS

EQUI-RESP-AFRICA programme is well placed to make a major contribution to addressing RDs within African countries. It will build on the stakeholder relationships and approaches to ‘pathways to impact’ that have already been established through NIHR RESPIRE Unit’s activities in South-East Asia, such as the use of pulse oximetry in child pneumonia in Bangladesh, where local researchers became the key technical partners to the Ministry of Health to redevelop all training materials at the national level. Moreover, the RESPIRE team also designed a national oxygen security survey for the Ministry of Health of Bangladesh as part of the COVID-19 response. Following those successful examples, EQUI-RESP-AFRICA will now collaborate with the ministries of health in African countries to plan the implementation of interventions against RDs and their risk factors, such as PR. We will also conduct CESI in a variety of ways and tailor it to local circumstances, *e.g.* by establishing PPI groups and national champions in stakeholder and community engagement. EQUI-RESP-AFRICA will also make important contributions to research training and capacity building in African institutions through in-person summer schools, e-learning platforms and education materials in digital health. We will leverage our close collaboration with RESPIRE in building capacity in preparedness for future emerging respiratory pathogens and standardising this across South-East Asia and Africa. Lastly, we will also aim to join and support the WHO’s Global Alliance against Respiratory Diseases. Our model will incorporate South-South support and training models. EQUI-RESP-AFRICA will build on existing excellent links with the WHO, UNICEF, the World Bank, ISoGH, and ministries of health to promote sustainability and long-term growth of its activities.

## Data Availability

**Data availability:** No data were generated during the preparation of this editorial.
